# *SPAG6* and *L1TD1* are transcriptionally regulated by DNA methylation in non-small cell lung cancers

**DOI:** 10.1186/s12943-016-0568-5

**Published:** 2017-01-05

**Authors:** Corinna Altenberger, Gerwin Heller, Barbara Ziegler, Erwin Tomasich, Maximilian Marhold, Thais Topakian, Leonhard Müllauer, Petra Heffeter, György Lang, Adelheid End-Pfützenreuter, Balazs Döme, Britt-Madeleine Arns, Walter Klepetko, Christoph C. Zielinski, Sabine Zöchbauer-Müller

**Affiliations:** 1 Department of Medicine I, Clinical Division of Oncology, Medical University of Vienna, Währinger Gürtel 18-20, 1090 Vienna, Austria; 2 Comprehensive Cancer Center, Medical University of Vienna, Vienna, Austria; 3 Department of Pathology, Medical University of Vienna, Vienna, Austria; 4 Department of Medicine I, Institute of Cancer Research, Medical University of Vienna, Vienna, Austria; 5 Division of Thoracic Surgery, Medical University of Vienna, Vienna, Austria; 6 Department of Thoracic Oncology and Tumor Biology, National Koranyi Institute of Pulmonology, Budapest, Hungary; 7 Department of Thoracic Surgery, Semmelweis University and National Institute of Oncology, Budapest, Hungary; 8 Department of Respiratory and Critical Care Medicine and Ludwig Boltzmann Institute for COPD, Otto-Wagner Hospital, Vienna, Austria

**Keywords:** Epigenetics, DNA methylation, Non-small cell lung cancer, *SPAG6*, *L1TD1*

## Abstract

**Background:**

DNA methylation regulates together with other epigenetic mechanisms the transcriptional activity of genes and is involved in the pathogenesis of malignant diseases including lung cancer. In non-small cell lung cancer (NSCLC) various tumor suppressor genes are already known to be tumor-specifically methylated. However, from the vast majority of a large number of genes which were identified to be tumor-specifically methylated, tumor-specific methylation was unknown so far. Thus, the major aim of this study was to investigate in detail the mechanism(s) responsible for transcriptional regulation of the genes *SPAG6* and *L1TD1* in NSCLCs.

**Methods:**

We analysed publically available RNA-sequencing data and performed gene expression analyses by RT-PCR. DNA methylation analyses were done by methylation-sensitive high-resolution melt analyses and bisulfite genomic sequencing. We additionally investigated protein expression using immunohistochemistry. Cell culture experiments included tumor cell growth, proliferation, viability as well as colony formation assays. Moreover, we performed xenograft experiments using immunodeficient mice.

**Results:**

We observed frequent downregulation of *SPAG6* and *L1TD1* mRNA expression in primary tumor ﻿(TU) samples compared to corresponding non-malignant lung tissue (NL) samples of NSCLC patients. We furthermore observed re-expression of both genes after treatment with epigenetically active drugs in most NSCLC cell lines with downregulated *SPAG6* and *L1TD1* mRNA expression. Frequent tumor-specific DNA methylation of *SPAG6* and *L1TD1* was detected when we analysed TU and corresponding NL samples of NSCLC patients. ROC curve analyses demonstrated that methylation of both genes is able to distinguish between TU and NL samples of these patients. Immunohistochemistry revealed a close association between *SPAG6*/*L1TD1* methylation and downregulated protein expression of these genes. Moreover, by performing functional assays we observed reduced cell growth, proliferation and viability of pCMV6-L1TD1 transfected NSCLC cells. In addition, reduced volumes of tumors derived from pCMV6-L1TD1 compared to pCMV6-ENTRY transfected NCI-H1975 cells were seen in a xenograft tumor model.

**Conclusions:**

Overall, our results demonstrate that *SPAG6* and *L1TD1* are tumor-specifically methylated in NSCLCs and that DNA methylation is involved in the transcriptional regulation of these genes. Moreover, in vitro as well as in vivo experiments revealed tumor-cell growth suppressing properties of *L1TD1* in NSCLC cells.

**Electronic supplementary material:**

The online version of this article (doi:10.1186/s12943-016-0568-5) contains supplementary material, which is available to authorized users.

## Background

DNA methylation (referred to as methylation) is a major epigenetic modification which regulates gene expression mainly by binding methyl-CpG binding proteins (MBDs) and their associated chromatin remodeling factors to DNA [1, 2]. It has been shown that methylation plays an important role in various molecular and cellular processes including embryonic development and genomic imprinting as well as in the pathogenesis of malignant diseases [3–7]. Methylation occurs by the covalent addition of a methyl group to the 5′ carbon of cytosines within CpG dinucleotides which is catalysed by DNA methyltransferases (DNMTs) [8, 9]. Unlike genetic changes, methylation may be reversed by DNMT inhibitors such as 5-aza-2′-deoxycytidine (5-AzadC) and 5-azacytidine (5-AzaC). A synergistic effect on re-expression of by methylation silenced genes by DNMT inhibitors in combination with histone deacetylase inhibitors like trichostatin A (TSA) was reported [10].

In non-small cell lung cancers (NSCLCs) numerous tumor suppressor genes (TSGs) are already known which are frequently methylated. Besides other epigenetic and genetic factors methylation may be responsible for transcriptional inactivation of these genes [11–15]. However, from the vast majority of ~500 genes which were identified to be tumor-specifically methylated in a genome-wide approach, tumor-specific methylation was unknown so far [12]. When we additionally analysed publically available microarray data in primary tumors (TU) compared to non-malignant lung tissue (NL) samples of NSCLC patients, we observed tumor-specifically downregulated expression of many of these genes [11, 16, 17]. An extensive PubMed search revealed that many of these genes are functionally uncharacterised in NSCLCs and in other cancer types. Based on all these observations, we selected the genes *SPAG6* (Sperm Associated Antigen 6) and *L1TD1* (LINE-1 Type Transposase Domain Containing 1) for detailed investigation. *SPAG6* is located in the chromosomal region 10p12.2 and is thought to be a cancer-testis antigen (CTA) [18]. CTAs represent a large family of cancer-associated antigens which are expressed in immunoprivileged tissues such as testis but were also detected in tumor tissues of various origins including lung cancer [19]. *SPAG6* is also expressed in normal lung tissues where it is associated with ciliary function [20]. It encodes a microtubule-associated protein which either functions as microtubule itself or binds to microtubules to form the cytoskeleton of the cell (www.pantherdb.org). There is increasing evidence that the expression of CTAs might be involved in tumorigenesis, however, so far there are no reports available about an involvement of *SPAG6* in malignant disease biology or cancer cell invasiveness [21]. *L1TD1* is located in the chromosomal region 1p31.3 where frequent loss of heterozygosity (LOH) was observed in NSCLCs [22]. This gene encodes a stem-cell specific RNA-binding protein required for self-renewal of human embryonic stem cells and for cancer cell proliferation [23]. Since the mechanism(s) of inactivation of both, *SPAG6* and *L1TD1*, were not studied in detail and their role in the pathogenesis of NSCLCs was unclear so far, we were interested to further investigate these genes.

Thus, we determined gene expression, methylation and re-expression of *SPAG6* and *L1TD1* in various NSCLC cell lines to elucidate if methylation is associated with the transcriptional inactivation of these genes. Moreover, we investigated tumor-specific methylation of these genes in a large number of NSCLC patients and compared these data as well as mRNA expression data with clinico-pathological characteristics of NSCLC patients. We also analysed protein expression of both genes in a subset of NSCLC patients and compared these results with *SPAG6* and *L1TD1* methylation. In addition, potential tumor-cell growth suppressing properties of these genes were investigated in in vitro studies and, for *L1TD1*, also in in vivo xenograft experiments.

Overall, we identified methylation as a mechanism involved in the regulation of transcriptional activity of *SPAG6* and *L1TD1* in NSCLCs. Furthermore, our results indicate that *L1TD1* functions as a tumor cell growth suppressor in NSCLC cells.

## Methods

### Publically available databases

IlluminaHiSeq RNA-sequencing (RNA-seq) data were obtained from “The Cancer Genome Atlas” (TCGA) database (https://cancergenome.nih.gov), Cancer Browser (https://genome-cancer.ucsc.edu) and from cBioPortal for Cancer Genomics (http://www.cbioportal.org) [24–28]. For analyses of single nucleotide variants (SNVs) and deletions of *SPAG6* and *L1TD1* lung adenocarcinoma (LUAD) and lung squamous cell carcinoma (LUSC) datasets were used. A summary of the clinico-pathological data of analysed patients is shown in Additional file 1: Table S2. For additional mRNA expression analyses, breast invasive carcinoma (BRCA), colon and rectum adenocarcinoma (COADREAD), head and neck squamous cell carcinoma (HNSC), kidney clear cell carcinoma (KIRC), liver hepatocellular carcinoma (LIHC) and prostate adenocarcinoma (PRAD) datasets were used (https://cancergenome.nih.gov). Data visualization of SNVs and homozygous and heterozygous deletions was performed using Caleydo software [29].

### Tumor cell lines and tissue samples

NSCLC cell lines A549 and NCI-H1993 were purchased from the American Type Culture Collection (ATCC), cell lines HCC827, NCI-H1650 and NCI-H1975 were kindly provided by Dr. Walter Berger (Institute of Cancer Research, Medical University of Vienna, Austria). Cell lines were maintained and grown as described [11]. Detailed descriptions of NSCLC and non-lung cancer cell lines are provided in Additional file 1: Table S1 and Table S4. Normal human bronchial epithelial cell (NHBECs) pellets were purchased from Promocell. For re-expression experiments tumor cells were treated either with the demethylating agent 5-AzadC alone or with a combination of 5-AzadC and the HDAC inhibitor TSA as described [30]. Untreated cells were used as controls. In addition, DNA of breast cancer (MCF-7, MDA-MB-453, MDA-MB-468, MDA-MB-231, BT20), colon cancer (HCT-15, HT29), ovarian cancer (SK-OV3, A2780), pancreatic cancer (AsPC-1, BxPC-3) as well as head and neck cancer cell lines (CAL27, FaDu) were provided by various members of the Medical University of Vienna, Austria. Primary TU and corresponding NL samples of 146 stage I-III NSCLC patients who underwent surgical resection of the tumor were collected and stored in liquid nitrogen until use. From 97 of these patients clinico-pathological characteristics including gender, age, histology, tumor stage, lymph node stage, disease stage, disease free survival (DFS) and overall survival (OS) were available. Moreover, from 35 of these patients formalin-fixed, paraffin embedded (FFPE) TU and NL samples were available and were used for immunohistochemistry. This study was approved by the local ethics committee.

### Methylation-sensitive high resolution melting analysis (MS-HRM)

Genomic DNA from NHBECs, 18 tumor cell lines and tissue samples of 146 NSCLC patients was isolated and modified by treatment with sodium bisulfite using EpiTect Bisulfite kit (Qiagen) [11]. The genomic sequences of *SPAG6* and *L1TD1* were obtained from ENSEMBL database (release 69). Primers were designed using Methyl Primer Express v1.0 software and are listed in the Additional file 1: Table S3. MS-HRM analyses were performed using a Rotor-Gene Q cycler (Qiagen) [31].

### Bisulfite genomic sequencing (BGS)

Sodium bisulfite treated genomic DNA was amplified (primer sequences listed in Additional file 1: Table S3). PCR products containing sequences which were also analysed by MS-HRM analyses were cloned using TOPO® TA Cloning® Kit for Sequencing (Invitrogen). Four clones per cell line were sequenced using M13 primers.

### Real-time reverse transcription PCR (RT-PCR)

Total RNA was isolated from NHBECs and from 5 NSCLC cell lines and reverse transcribed using OmniScript Reverse Transcriptase Kit (Qiagen) [11]. Expression of *SPAG6* was determined using Taqman Gene Expression Assays Hs00542625_m1 (*SPAG6*) and Hs03929097_g1 (*GAPDH*) in a StepOne cycler (Applied Biosystems) and expression of *L1TD1* was determined using Qiagen’s QuantiTect® SYBR Green PCR Kit (primer sequences see Additional file 1: Table S3). The ΔΔCt method was used to calculate differences in gene expression [32].

### Immunohistochemistry (IHC)

Tissue microarrays with 1 mm cores and 5 μm sections from FFPE TU and NL samples were used for protein expression analyses of SPAG6 and L1TD1. Samples were stained with the rabbit polyclonal antibodies HPA038440 (SPAG6 1:100, Sigma Aldrich) and HPA028501 (L1TD1 1:100, Sigma Aldrich). Results of IHC were scored as no staining (−), weak staining (−/+), moderate staining (1+) or strong staining (≥2+). For comparison with methylation results patients whose TU showed no or weak staining were grouped as “negative by IHC” while patients whose TU showed moderate or strong staining were grouped as “positive by IHC” as reported previously [12].

### Cell transfection

NCI-H1975 cells were transfected with pCMV6-ENTRY (PS100001, Origene) and pCMV6-L1TD1 (RC219014, Origene) expression vectors using Lipofectamine® LTX reagent (Invitrogen). Additionally, a pCMV6-GFP vector was constructed by subcloning the GFP coding sequence into existing pCMV6-ENTRY vector (primer sequences see Additional file 1: Table S3). Stably transfected NCI-H1975 cells were selected by G418 treatment (Invitrogen) and transfection efficacy was analysed by RT-PCR and Western blotting [11].

### Immunoprecipitation and Western blot (IP-WB)

IP was used to enrich L1TD1 protein after transfection with pCMV6-L1TD1 expression vector of NCI-H1975 cells. Transfected cells were lysed in Pierce^®^ IP Lysis Buffer (Thermo Scientific) containing PhosSTOP and EDTA-free inhibitors (Roche). IP was carried out using L1TD1 Antibody (HPA028501, Sigma-Aldrich), normal rabbit IgG (#2729, Cell Signaling) and Protein A/G PLUS-Agarose beads (Santa Cruz). Western blot analysis was performed as described [11].

### Cell proliferation assay

The xCELLigence Real-Time Cellular Analysis (RTCA) System (Roche) was used to measure cell proliferation in real-time. Stably transfected NCI-H1975 cells were seeded in triplicates and cell proliferation was monitored [11].

### Cell viability assay

Stably transfected NCI-H1975 cells were seeded in triplicates in 96-well plates and incubated with CellTiter™ Blue reagent (Promega). Fluorescence was measured repeatedly using a TriStar microplate reader (Berthold Technologies) [11].

### Colony formation assay

After selection of stably transfected NCI-H1975 cells, cells were grown until differences in colony-forming abilities were detected. Before imaging cells were stained with 0.05% crystal violet dye.

### Xenograft tumor model

All animal experiments were approved by and carried out according to guidelines from the Austrian Federal Ministry of Science, Research and Economy and the Animal Ethics Committee of the Medical University of Vienna. For the xenograft tumor model female NSG JAX (NOD.Cg-*Prkdc* ^*scid*^ *Il2rg* ^*tm1Wjl*^ */SzJ*) mice (NSG mice) were purchased from Charles River Laboratories and housed under pathogen-free conditions in the animal facility of the Medical University of Vienna. 5x10^5^ NCI-H1975 cells stably transfected with *L1TD1* expression vector or with empty control vector were injected subcutaneously into the left respectively right flanks of 4 17 weeks old NSG mice. Animals were checked for overall health regularly and tumor size was measured using a digital caliper. Tumor volume was calculated using the standard formula (length x width^2^)/2 [33]. At the end of the experiment mice were sacrificed by cervical dislocation and dissected tumors were snap frozen and processed for RT-PCR analyses.

### Statistical analyses

Wilcoxon signed rank tests and receiver operating characteristic (ROC) curve analyses were performed to determine methylation differences between TU and NL samples obtained by MS-HRM analyses. Spearman correlation was determined to investigate correlation between methylation and expression data. To calculate statistical differences between the tumor volumes of xenografts two-way ANOVA test was performed. These analyses were performed using GraphPad Prism 6 software with two-sided p-values < 0.05 considered as statistically significant. MS-HRM data were compared with clinico-pathological characteristics of NSCLC patients. Chi^2^ tests/ Fisher’s exact tests were applied to calculate differences between groups and t-tests were used to calculate differences between means. For methylation analyses T/N ratios were calculated and a ratio of > 1.5 was used as cut off for samples to be considered as methylated. Survival analyses of patients were performed using log rank testing. Cox regression was used for univariate analyses on overall survival and to calculate hazard ratios with 95% confidence intervals. A p-value < 0.05 was considered as statistically significant. Analyses were performed using the statistics software PASW (version 18). Kaplan-Meier plots were generated using Kaplan-Meier plotter [34]. The cut-off values for “low” and “high” *SPAG6* as well as *L1TD1* mRNA expression were automatically defined by KM plotter software.

## Results

### Downregulation of *SPAG6* and *L1TD1* mRNA expression in NSCLCs and in other tumor entities

**Fig. 1:**
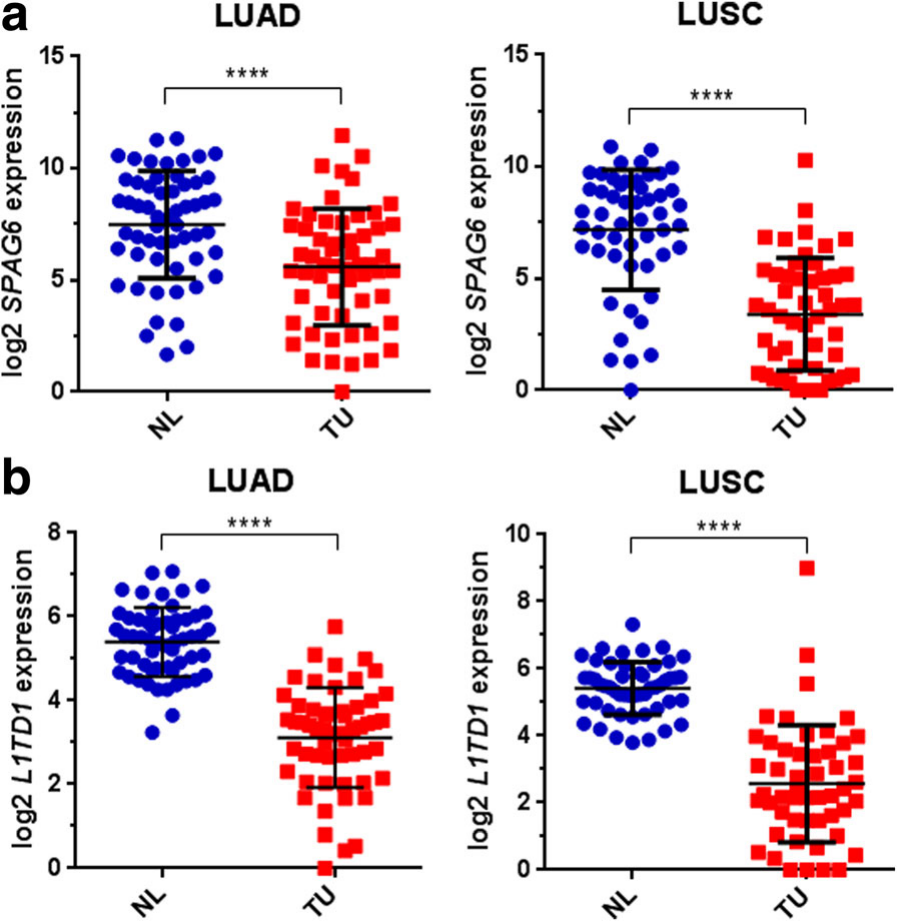
*SPAG6* and *L1TD1* mRNA expression in TU and corresponding NL samples of NSCLC patients in TCGA datasets. Comparison of *SPAG6* **a** and *L1TD1* **b** mRNA expression in TU and NL samples of 57 adenocarcinoma (LUAD dataset) and 51 squamous cell carcinoma patients (LUSC dataset), respectively. Normalized log2 expression values are shown, ****p < 0.0001. Only patients with matched TU and NL samples were used for this analysis. TU, primary tumor sample; NL, normal lung tissue sample

We analysed publically available RNA-seq data from TCGA database to investigate *SPAG6* and *L1TD1* mRNA expression in NSCLC patients. By comparison of RNA-seq expression values in TU and corresponding NL samples of LUAD and LUSC datasets, we observed a statistically significant downregulation of *SPAG6* and *L1TD1* mRNA expression in lung adenocarcinomas (*p* < 0.0001, respectively; Fig. 1a) as well as in lung squamous cell carcinomas (*p* < 0.0001, respectively; Fig. 1b). In addition, we analysed mRNA expression of these genes also in tumors of other histologies by the use of different datasets from the TCGA database [24]. Compared to non-malignant tissues we observed statistically significant downregulation of *SPAG6* in breast carcinomas (*p* = 0.0039), colorectal carcinomas (*p* < 0.0001), head and neck carcinomas (*p* = 0.0030), kidney clear cell carcinomas (*p* < 0.0001) and in hepatocellular carcinomas (*p* = 0.0046), but not in prostate carcinomas (Additional file 2: Figure S1). In contrast, *L1TD1* expression was significantly downregulated in breast carcinomas (*p* = 0.0253), colorectal carcinomas (*p* = 0.0224) and in prostate carcinomas (*p* < 0.0001), but not in head and neck carcinomas, kidney clear cell carcinomas and in hepatocellular carcinomas (Additional file 2: Figure S1).

### Transcriptional silencing of *SPAG6* and *L1TD1* in cancer cell lines is caused by methylation of *SPAG6* and *L1TD1*

**Fig. 2:**
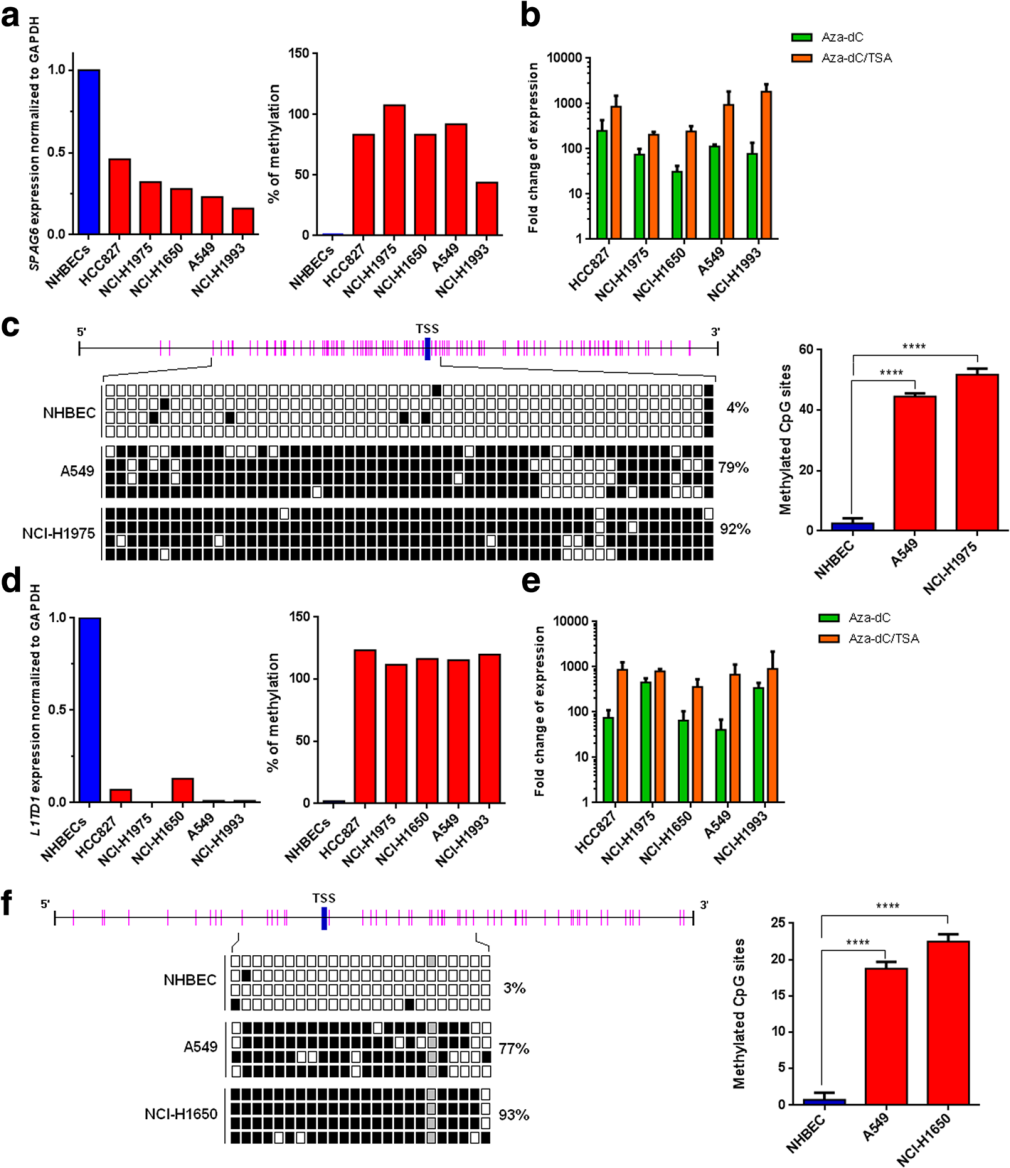
Expression and methylation analyses of *SPAG6* and *L1TD1* in NHBECS and in NSCLC cell lines. **a** By RT-PCR *SPAG6* mRNA was found to be downregulated in NSCLC cell lines but not in NHBECs. In contrast, *SPAG6* methylation was observed in all NSCLC cell lines with downregulated mRNA expression but not in NHBECs by MS-HRM analysis. **b** *SPAG6* methylated cells were treated with 5-AzadC alone or with a combination of 5-AzadC and TSA. RT-PCR analyses revealed upregulation of *SPAG6* expression after drug treatment. Fold changes of gene expression in treated cells compared to untreated cells are shown. **c** Results from BGS of a part of the *SPAG6* 5′ region in NHBECs, A549 and NCI-H1975 cells are shown. 56 CpG sites (indicated as pink bars) were analysed for *SPAG6* methylation. TSS, transcription start site, black squares indicate methylated CpG sites, white squares unmethylated CpG sites. Analyses of BGS results of the *SPAG6* 5′ region showed statistically significant differences of methylated CpG sites between NHBCEs and NSCLC cells (****p < 0.0001). **d** *L1TD1* mRNA was found to be downregulated in NSCLC cell lines but not in NHBECs by RT-PCR. By MS-HRM analysis *L1TD1* methylation was detected in all NSCLC cell lines with downregulated mRNA expression but not in NHBECs. **e** *L1TD1* methylated cells were treated with epigenetically active drugs. RT-PCR analyses revealed upregulation of *L1TD1* expression after drug treatment. Fold changes of gene expression in treated cells compared to untreated cells are shown. **f** Results from BGS of a part of the *L1TD1* 5′ region in NHBECs, A549 and NCI-H1650 cells are shown. Twenty-four CpG sites were analysed for *L1TD1* methylation. TSS, transcription start site, black squares indicate methylated CpG sites, white squares unmethylated CpG sites and grey squares intermediate methylation. Analysis of BGS results of the *L1TD1* 5′ region showed statistically significant differences of methylated CpG sites between NHBCEs and NSCLC cells (****p < 0.0001)

We additionally determined mRNA expression of these genes in NSCLC cells as well as in NHBECs. While *SPAG6* and *L1TD1* mRNA expression was observed in NHBECs, expression of these genes was decreased in cells of all 5 NSCLC cell lines analysed (Fig. 2a and d). In order to investigate if downregulated *SPAG6* and *L1TD1* expression in NSCLC cells may be caused by methylation, we developed MS-HRM assays to analyse methylation of the 5′- promoter regions of these genes. *SPAG6* and *L1TD1* were found to be methylated in cells of all 5 NSCLC cell lines, but not in NHBECs (Fig. 2a and d).

Moreover, we treated cells of the 5 NSCLC cell lines found to be *SPAG6* and *L1TD1* methylated with either 5-AzadC alone and with a combination of 5-AzadC and TSA. We observed an upregulation of *SPAG6* as well as of *L1TD1* mRNA expression after treatment with epigenetically active drugs compared to untreated cells by RT-PCR (Fig. 2b and e). To confirm data obtained by MS-HRM analyses, we also performed bisulfite genomic sequencing (BGS) of parts of the 5′-regions of these genes in cells of selected NSCLC cell lines and in NHBECs. While most of the 56 CpG sites analysed were found to be *SPAG6* methylated in A549 (79%) and in NCI-H1975 (92%) cells, only a few CpG sites were methylated in NHBECs (4%, Fig. 2c). Differences in the percentage of *SPAG6* methylated CpG sites between NSCLC cells and NHBECs were statistically significant (*p* < 0.0001). *L1TD1* was methylated in 77% and 93% of 24 CpG sites analysed in A549 and in NCI-H1975 cells, however, in NHBECs only 3% of CpG sites were methylated for this gene (Fig. 2f). Differences of *L1TD1* methylation between NSCLC cell lines and NHBECs were statistically significant (*p* < 0.0001).

Moreover, we determined methylation of *SPAG6* and *L1TD1* in 5 breast cancer, 2 colon cancer, 2 ovarian cancer, 2 pancreatic cancer as well as 2 head and neck cancer cell lines. All these tumor cell lines were found to be *SPAG6* and *L1TD1* methylated with percentages of methylation ranging between 71% (SK-OV3 cells) and 98% (FaDu cells) for *SPAG6* methylation and between 88% (BxPC-3 cells) and 100% (FaDu cells) for *L1TD1* methylation (Additional file 1: Table S4).

### *SPAG6* and *L1TD1* are tumor-specifically methylated in NSCLC patients

**Fig. 3:**
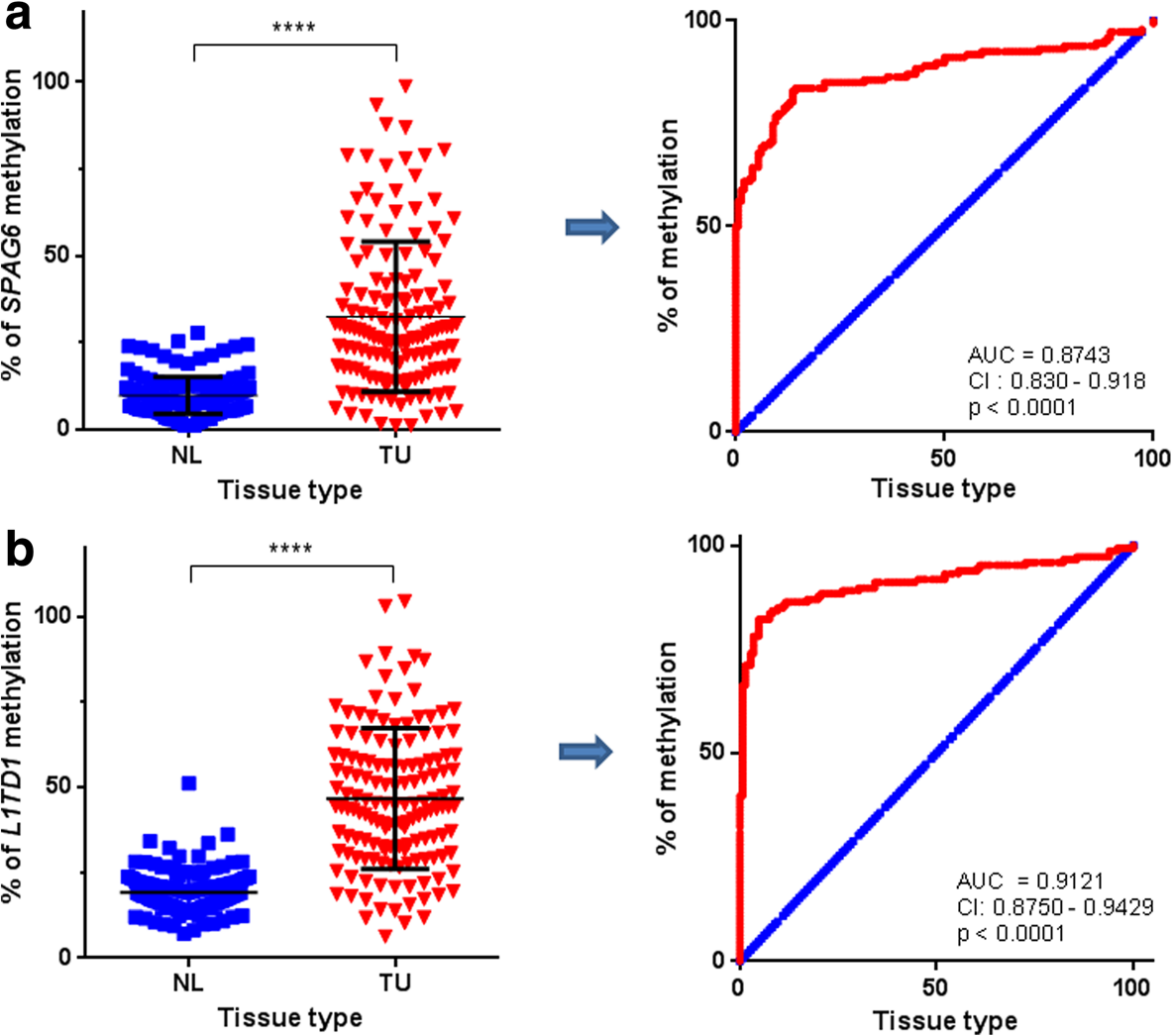
*SPAG6* and *L1TD1* methylation in NSCLC patients. Comparison of *SPAG6* **a** and *L1TD1* **b** methylation percentages in TU and NL samples of 146 NSCLC patients with each dot representing methylation percentages in an individual sample revealed statistically significant differences (****p < 0.0001). ROC curve analyses demonstrated that the extent of *SPAG6* **a** and *L1TD1* **b** methylation is able to distinguish between TU and NL samples. Blue line, reference line; red line, methylation of *SPAG6*; AUC, area under the curve; CI, confidence interval; TU, primary tumor sample; NL, normal lung tissue sample

We also investigated *SPAG6* and *L1TD1* methylation in TU and corresponding NL samples of 146 stage I-III NSCLC patients by MS-HRM analyses. Differences in *SPAG6* and *L1TD1* methylation between TU and NL samples were statistically significant (*p* < 0.0001, respectively) demonstrating that both genes are tumor-specifically methylated (Fig. 3a and b). Furthermore, ROC curve analyses of methylation results revealed that *SPAG6* as well as *L1TD1* methylation is able to distinguish TU from NL samples of NSCLC patients (*p* < 0.0001, respectively; Fig. 3a and b). Moreover, for each patient T/N ratios of *SPAG6* and *L1TD1* methylation were calculated as the % of methylation in the primary TU/ % of methylation in the corresponding NL sample [12]. Considering patients with a T/N ratio ≥ 1.5 as methylated, 79% of them were *SPAG6* methylated and 81% of them were *L1TD1* methylated, respectively. In addition, T/N ratios of *SPAG6* and *L1TD1* methylation were used to compare methylation results with clinico-pathological characteristics of the patients. No statistically significant associations were observed.

However, we observed a statistically significant shorter OS of patients with squamous cell carcinoma subtype and low *SPAG6* mRNA expression levels as well as of patients with adenocarcinoma subtype and low *L1TD1* mRNA expression levels (Additional file 3: Figure S2; https://cancergenome.nih.gov/).

### *SPAG6* and *L1TD1* SNVs and deletions in NSCLC patients

To investigate if other mechanisms besides methylation are involved in transcriptional regulation of *SPAG6* and *L1TD1*, we analysed SNVs as well as homozygous and heterozygous deletions in LUAD and LUSC SNP and aCGH datasets of NSCLC patients. In lung adenocarcinoma patients only 3% had *SPAG6* SNVs*.* While no homozygous *SPAG6* deletions were detected, 22% of these patients showed *SPAG6* heterozygous deletions (Additional file 4: Figure S3). In lung squamous cell carcinoma patients *SPAG6* SNVs were seen in only 2%. Homozygous *SPAG6* deletions were detected in only 1% and heterozygous *SPAG6* deletions were found in 40% of these patients. Similar frequencies of SNVs and deletions were found for *L1TD1* (Additional file 4: Figure S3).

### Frequent loss of SPAG6 and L1TD1 protein expression in NSCLC patients

**Fig. 4:**
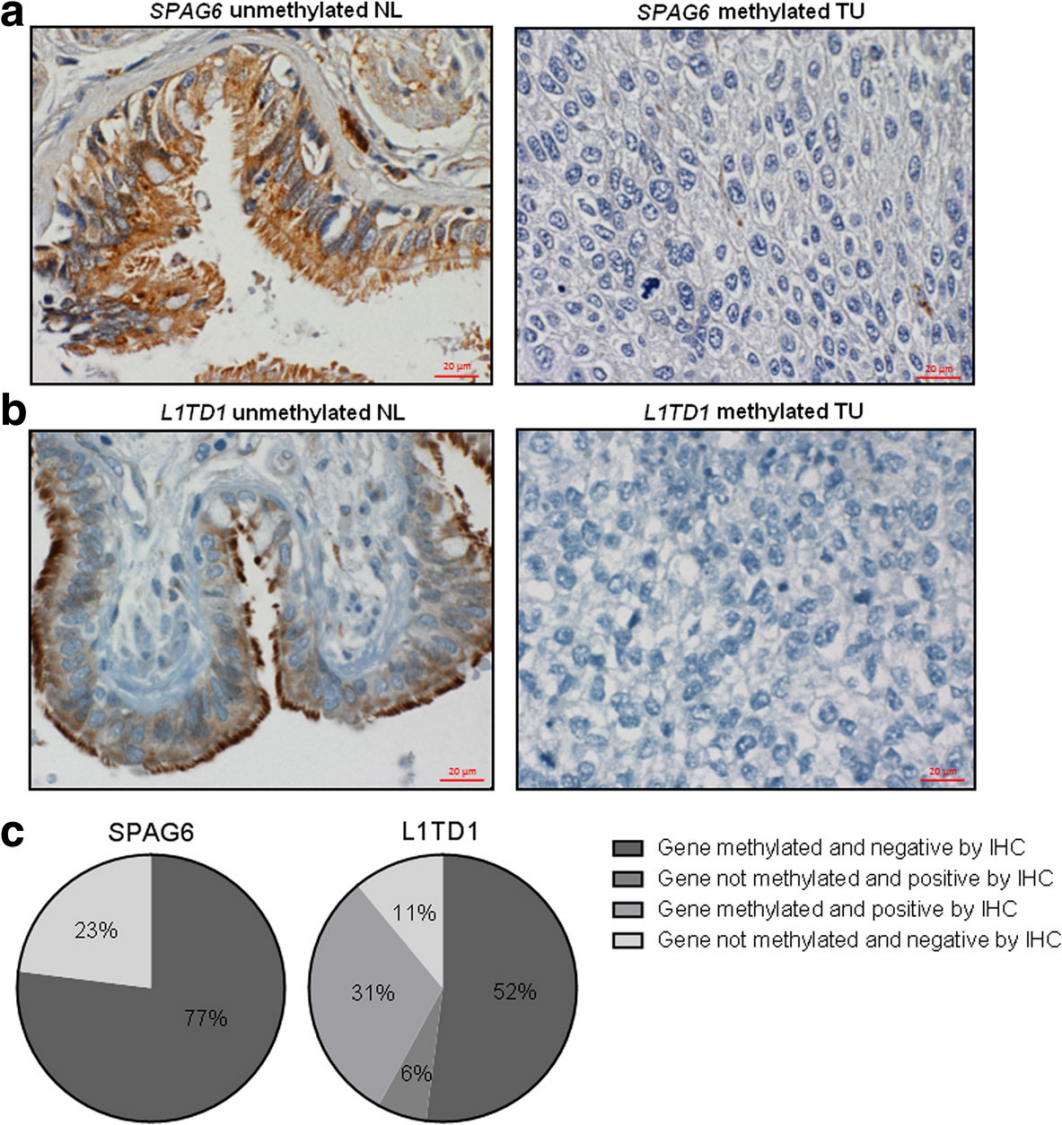
Protein expression of SPAG6 and L1TD1 in NSCLC patients. Representative IHC stainings of NL and TU samples are shown. In unmethylated NL samples cytoplasmic expression of SPAG6 **a** and L1TD1 **b** was observed in bronchial and bronchiolar epithelial cells. In *SPAG6* **a**, respectively, *L1TD*1 **b** methylated TU samples neither SPAG6 nor L1TD1 expression was observed in tumor cells. **c** The comparison between SPAG6 and L1TD1 protein expression and *SPAG6* or *L1TD1* methylation by T/N methylation ratios and IHC groupings of 35 TU samples is shown

To compare *SPAG6* and *L1TD1* methylation with their protein expression in NSCLC patients, we performed IHC of FFPE TU and NL samples of a subgroup of 35 NSCLC patients. These samples were also analysed for *SPAG6* and *L1TD1* methylation by MS-HRM. SPAG6 and L1TD1 protein expression was observed in bronchial and bronchiolar epithelial cells of NL samples. L1TD1 protein expression was also detected in alveolar epithelial cells. However, no or only weak SPAG6 staining was detected in 60% and in 40% of the TU samples, respectively. Moreover, no L1TD1 protein expression was detected in 51% and only weak staining in 11% of the TU samples. Moderate or strong staining was seen in 20% and in 17% of the TU specimens, respectively. Representative stainings of TU and NL samples are shown in Fig. 4. When we compared *SPAG6* and *L1TD1* methylation with their protein expression in TU samples, we observed downregulated SPAG6 protein expression in 77% of *SPAG6* methylated TU samples and downregulated L1TD1 protein expression in 52% of *L1TD1* methylated TU samples, respectively (Fig. 4c).

### Effects of *SPAG6* and *L1TD1* expression on growth, proliferation, viability and colony-forming abilities of NSCLC cells

**Fig. 5:**
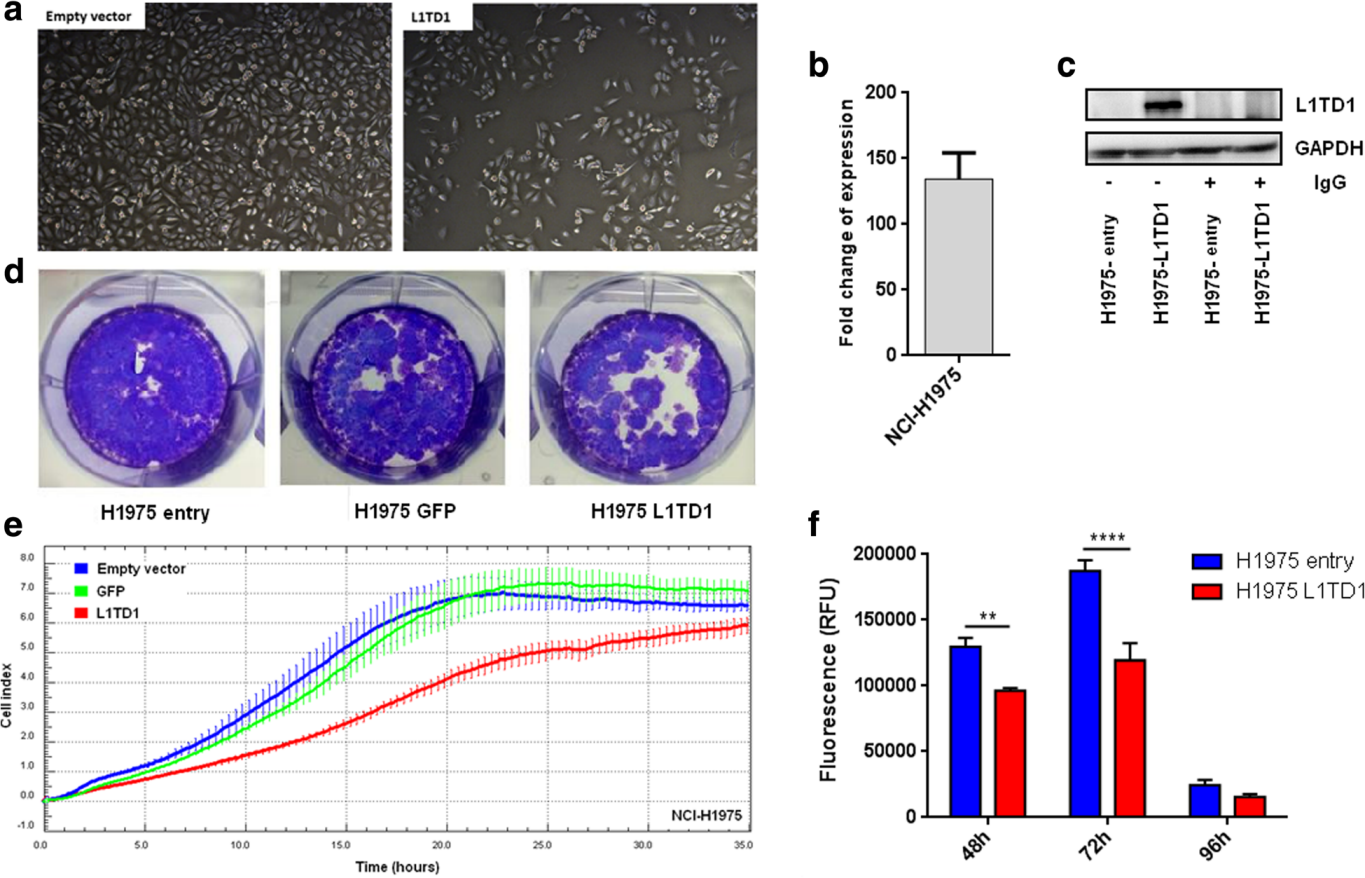
Growth, proliferation, colony-forming ability and viability of pCMV6-L1TD1 and pCMV6-ENTRY transfected NCI-H1975 cells. **a** Reduced growth of pCMV6-L1TD1 compared to pCMV6-ENTRY transfected cells was observed. Impact of G418 treatment was monitored using untransfected cells. **b** Fold change of *L1TD1* mRNA expression in pCMV6-L1TD1 compared to pCMV6-ENTRY transfected NCI-H1975 cells normalized to *GAPDH* expression is shown. **c** L1TD1 protein expression in pCMV6-L1TD1 and pCMV6-ENTRY transfected cells was analysed by IP-WB using normal rabbit IgG as non-specific IP control and GAPDH as loading control. **d** Reduced colony formation and **e** reduced cell proliferation of pCMV6-L1TD1 compared to pCMV6-ENTRY transfected cells was found. Each cell line was plated in triplicates. Error bars indicate standard deviations. **f** Reduced cell viability of pCMV6-L1TD1 compared to pCMV6-ENTRY transfected cells was observed. Results from CellTiter™ Blue assay at different time points are shown. Experiments were performed in triplicates (**p < 0.05, ** **p < 0.0001)

To investigate potential tumor cell - growth suppressing properties of *L1TD1*, we stably transfected NCI-H1975 cells with a pCMV6-L1TD1 expression vector, with a pCMV6-ENTRY control vector as well as with a constructed pCMV6-GFP vector. We found a strong reduction in tumor cell growth of pCMV6-L1TD1 compared to pCMV6-ENTRY transfected cells (Fig. 5a). Overexpression of *L1TD1* in pCMV6-L1TD1 transfected cells was confirmed by RT-PCR (Fig. 5b) and IP-WB (Fig. 5c). Moreover, we analysed tumor cell proliferation in a time-dependent manner and observed a reduced proliferation rate of pCMV6-L1TD1 compared to pCMV6-ENTRY and to pCMV6-GFP transfected cells (Fig. 5F). In addition, we found a reduced colony-forming ability and tumor cell viability of pCMV6-L1TD1 transfected cells compared to control cells (Fig. 5d, e). Differences in tumor cell viability between pCMV6-L1TD1 and pCMV6-ENTRY transfected cells were statistically significant after 48 (*p* = 0.0064) and 72 h (*p* < 0.0001), while differences between pCMV6-L1TD1 and pCMV6-GFP transfected cells did not reach statistical significance. Similar in vitro experiments were performed for *SPAG6*. However, no impact of ectopic SPAG6 expression on tumor cell growth, proliferation, viability or colony-forming abilities were seen (data not shown).

### *L1TD1* reduces tumor growth in vivo

**Fig. 6:**
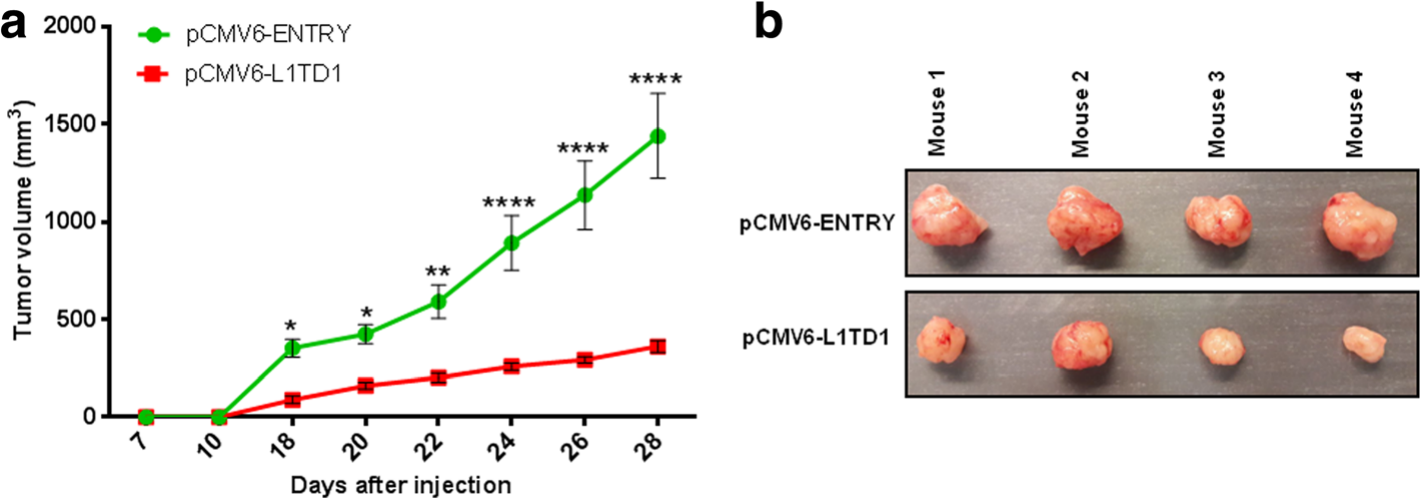
Xenograft tumor model. **a** Reduced volumes of xenograft tumors derived from pCMV6-L1TD1 compared to pCMV6-ENTRY transfected NCI-H1975 cells were observed and are shown as mean with SEM (**p = 0.0078 respectively *p* = 0.0075, ***p = 0.0003, ****p < 0.0001). **b** Differences in the size of xenograft tumors were confirmed after dissection. Upper panel: xenograft tumors expressing pCMV6-ENTRY, lower panel: xenograft tumors expressing pCMV6-L1TD1

Because in vitro experiments suggested tumor-cell growth suppressing properties of *L1TD1*, we additionally performed in vivo studies using immunodeficient mice. Therefore, pCMV6-L1TD1 and pCMV6-ENTRY transfected cells were subcutaneously injected into the ventral flanks of NSG mice. Ten days after injection 4/4 mice had measurable lesions in both flanks. From day 18 after injection on statistically significant differences in tumor volumes between the two groups were observed (Fig. 6a). At day 28 after injection the experiment was terminated and tumors were dissected. Size measurement of dissected tumors confirmed size differences of tumors derived from pCMV6-L1TD1 and from pCMV6-ENTRY transfected cells which were seen during the experiment (Fig. 6b). Expression of *L1TD1* in tumors derived from pCMV6-L1TD1 transfected NCI-H1975 cells was confirmed by RT-PCR (Additional file 5: Figure S4).

## Discussion

In a recent study, we performed a genome-wide screen for CpG island methylation in NSCLC patients and identified more than 400 tumor-specifically methylated genes. Two of them, *SPAG6* and *L1TD1*, were selected for detailed investigation of gene expression, gene-specific methylation and potential tumor-cell growth suppressing properties in NSCLCs. So far, only little information about a potential impact of these 2 genes on the development of lung cancer was available.

*SPAG6* regulates proliferation and differentiation of certain cell types and belongs to the family of CTAs [35, 36]. Normally CTAs are expressed by gametes and trophoblasts but are aberrantly expressed in a variety of tumors [21]. Their transcriptional activity is mainly regulated by epigenetic modifications including methylation and histone acetylation [21, 37]. The role of CTAs in germ line as well as in tumor tissues is poorly understood, however, since germ and cancer cells share certain characteristics including immortalization, invasion and migration, an involvement of CTAs in the development of different tumor types is suggested [37].

*L1TD1* is involved in the regulation of self-renewal and pluripotency of human embryonic stem cells. It is highly expressed in medulloblastoma cells where it is associated with cell viability, chemotherapeutic drug resistance and stem cell-like properties [38].

To determine a potential role of *SPAG6* and/or *L1TD1* in various malignancies, we analysed RNA-seq datasets from the TCGA database. While tumor-specific downregulation of *SPAG6* mRNA expression was observed in all tumor types investigated except hepatocellular and prostate carcinomas, downregulated *L1TD1* mRNA expression was found in NSCLCs, breast, colorectal and prostate carcinomas but not in head and neck, kidney and hepatocellular carcinomas. Overall, these data suggest that deregulated expression of *SPAG6* and *L1TD1* may play a role not only in the pathogenesis of NSCLCs but also in tumors of other entities and that expression of these 2 genes differs between certain tumor types.

Besides other mechanisms, methylation is involved in the regulation of transcriptional gene activity [1, 2]. In NSCLCs, many TSGs are already known which are frequently inactivated by methylation [11, 13, 15, 39]. To determine if downregulation of *SPAG6* and *L1TD1* may be caused by methylation, we performed gene-specific approaches to detect mRNA/protein expression and methylation of these genes in NSCLC cell lines and in primary TU and NL samples from NSCLC patients. Indeed, we found a correlation between downregulated *SPAG6* and *L1TD1* mRNA expression and methylation of these genes in all NSCLC cell lines analysed. Treatment of these cells with epigenetically active drugs which inhibit DNA methyltransferases and histone deacetylases resulted in upregulated expression of both genes. In addition, we found an association between *SPAG6* and *L1TD1* methylation and loss of SPAG6 and L1TD1 protein expression when we performed IHC of FFPE tissue samples from NSCLC patients. While most of the *SPAG6* or *L1TD1* methylated TU samples did not express these proteins, not methylated TU samples mostly expressed SPAG6 or L1TD1. However, these results did not reach statistical significance, probably because of the low sample number available for these analyses and the fact that tissue microarrays only represented small parts of the primary tumor samples. Our hypothesis that methylation is one of the mechanisms responsible for inactivation of these genes is further supported by the fact that SNVs and homozygous deletions of *SPAG6* and *L1TD1* were rarely detected in LUAD and LUSC SNP and in aCGH datasets of NSCLC patients. However, other mechanisms including deregulation of histone modifications and non-coding RNAs may also contribute to the transcriptional regulation of these genes. All these findings indicate that methylation is indeed involved in the transcriptional regulation of *SPAG6* and *L1TD1* in NSCLCs.

Methylation of certain TSGs in NSCLCs is tumor-subtype specific [40]. While for instance *APC* and *CDH13* methylation was detected more frequently in lung adenocarcinomas compared to lung squamous cell carcinomas, *p16* methylation was detected more frequently in lung squamous cell carcinomas than in lung adenocarcinomas [40, 41]. By comparing *SPAG6* and *L1TD1* methylation with histological classification of our tumor samples, we did not find a difference between the frequencies of methylation of these genes in lung adenocarcinomas or lung squamous cell carcinomas. These observations suggest that methylation of *SPAG6* and *L1TD1* is a common feature in all histological subtypes of NSCLCs. Methylation of several genes was shown to be associated with shorter survival of NSCLC patients (e.g. *APC*, *CDH1*, *DAPK* or *p16*) [13, 42, 43]. In our study, we did not find a correlation between *SPAG6* or *L1TD1* methylation and OS as well as DFS of NSCLC patients or any other clinico-pathological characteristic of these patients. However, analyses of gene expression microarray data indicate that low *SPAG6* expression is associated with a shorter OS of lung squamous cell carcinoma patients and low *L1TD1* expression with a shorter OS of lung adenocarcinoma patients. Nevertheless, these findings need to be studied in larger patient cohorts.

TSGs are characterised by a variety of molecular features including frequent CpG island methylation, downregulated expression and that they are often located in regions of LOH. Since *SPAG6* and *L1TD1* were found to be frequently methylated and downregulated in NSCLC cells and are located in chromosomal regions where frequent LOH was observed, we hypothesized that these genes may have tumor-cell growth suppressing properties. Indeed, in vitro experiments showed reduced cell growth, proliferation, viability and colony-forming abilities of pCMV6-L1TD1 transfected cells suggesting that it may be a potential TSG in NSCLCs. Because of these encouraging results and to further support our hypothesis that *L1TD1* has tumor-cell growth suppressing properties, we additionally performed xenograft experiments to investigate the growth of tumors induced by L1TD1-overexpressing and wildtype NSCLC cells. Interestingly, we observed significantly smaller tumors induced by pCMV6-L1TD1 compared to pCMV6-ENTRY transfected NCI-H1975 cells indicating that *L1TD1* indeed has tumor-cell growth suppressing properties. Based on the literature this is the first report which describes a potential impact of *L1TD1* expression in the pathogenesis of NSCLCs. However, further studies are necessary to elucidate molecular mechanisms affected by *L1TD1* in NSCLC cells.

Although it was reported that SPAG6 is involved in proliferation and differentiation of neuronal progenitor cells, in our in vitro studies ectopic SPAG6 expression did not affect the behavior of NSCLC cells [35, 36]. Since no reports are currently available about a potential role of *SPAG6* in malignant diseases further studies are necessary to determine cell-type specific functions of *SPAG6* and to elucidate if besides tumor-specific methylation other functions of *SPAG6* may be involved in the pathogenesis of NSCLCs.

## Conclusion

Our results demonstrate that tumor-specific methylation of *SPAG6* and *L1TD1* is a frequently occurring event in NSCLCs and they suggest that methylation plays an important role in the transcriptional regulation of these genes. Protein expression of both genes was frequently downregulated in primary NSCLCs. In addition, or findings indicate that methylation of these genes may be of relevance not only in NSCLCs but also in other malignancies. Moreover, in vitro and in vivo experiments showed that *L1TD1* has tumor-cell growth suppressing properties in NSCLC cells. Taken together we identified methylation as a potential mechanism for frequent downregulation of *SPAG6* and *L1TD1* in NSCLCs and suggest a putative role of *L1TD1* in tumor cell development.

## Additional files

Additional file 1: Table S1. Description of NSCLC cell lines used in this study. Information about histology, origin and disease stage of donors was obtained from ATCC catalogue (https://www.lgcstandards-atcc.org). *EGFR*, *KRAS* and *TP53* mutational status and *MET* amplification according to supplementary references (1–3). *activating *EGFR* mutation in exon 19 (E746-E749 del), **activating *EGFR* mutation in exon 21 (L858R). N/A, not available; wt, wildtype; mut, mutated. **Table S2.** Clinico-pathological characteristics of 983 NSCLC patients. Overview of gender, histology, stage of disease and ethnicity of NSCLC patients obtained from TCGA database and used for mutation and copy number changes analyses of SPAG6 and L1TD1 is shown. ADC, adenocarcinoma; SCC, squamous cell carcinoma. Clinical data based on Caleydo software version 16/04/14. **Table S3.** Primer sequences. Summary of oligonucleotide sequences used for mRNA expression, MS-HRM, BGS analyses and construction of pCMV6-GFP expression vector. Y, random integration of C or T in fwd primer; R, random integration of G or A in rev primer. **Table S4.** Methylation of *SPAG6* and *L1TD1* in tumor cells of other tumor types. *Morphology, histology and origin of cell lines according to ATCC catalogue (https://www.lgcstandards-atcc.org). Percentage of methylation was calculated as described previously (4). (DOCX 33 kb)
Additional file 2: Figure S1.
*SPAG6* and *L1TD1* mRNA expression in different datasets of TCGA database. *SPAG6* and *L1TD1* mRNA expression was analysed using IlluminaHiSeq RNAseq data from TCGA database. Datasets LUAD and LUSC (lung), BRCA (breast), COADREAD (colorectal), HNSC (head and neck), KIRC (kidney), LIHC (liver) and PRAD (prostate) were analysed. Normalized log2 mRNA expression values are shown. Each dot represents a single sample. (TIF 176 kb)
Additional file 3: Figure S2. Impact of *SPAG6* and *L1TD1* mRNA expression on OS of NSCLC patients. (A) A shorter OS of squamous cell carcinoma patients with low *SPAG6* mRNA expression (N = 155) compared to high *SPAG6* mRNA expression (N = 267) was observed. (B) Adenocarcinoma patients with low *L1TD1* mRNA expression (N = 138) showed a shorter OS compared to adenocarcinoma patients with high *L1TD1* mRNA expression (N = 350). Gene expression microarray datasets (Affymetrix IDs 210032_s_at and 219955_at) were analysed and Kaplan-Meier plots were generated using all datasets and default settings of KM plotter. The cut-off values for “low” and “high” *SPAG6* and *L1TD1* mRNA expression were automatically defined by KM plotter software (Version 2013). (TIF 50 kb)
Additional file 4: Figure S3.
*SPAG6* and *L1TD1* SNVs and deletions in NSCLC patients. TCGA LUAD and LUSC datasets were analysed with Caleydo software (version April 2014). Mutation of *TP53* was used to demonstrate reliability of TCGA data analysis. ADC, adenocarcinoma patients; SCC, squamous carcinoma patients. (TIF 90 kb)
Additional file 5: Figure S4.
*L1TD1* mRNA expression in xenograft tumors. Expression of *L1TD1* in 4 xenografts derived from pCMV6-L1TD1 transfected NCI-H1975 cells was confirmed by RT-PCR. *GAPDH* was used as housekeeping gene to normalize mRNA expression of *L1TD1. (TIF 17 kb)*

## Abbreviations

5-AzaC: 5-azacytidine
5-AzadC: 5-aza-2′-deoxycytidine
aCGH: Microarray Comperative Genomic Hybridization
BGS: Bisulfite genomic sequencing
BRCA: Breast invasive carcinoma
COADREAD: Colon and rectum adenocarcinoma
CTA: Cancer-testis antigen
DFS: Disease free survival
DNMT: DNA methyltransferases
FFPE: Formalin-fixed, paraffin embedded samples
HDAC: Histone deacetylase
HNSC: Head and neck squamous cell carcinoma
IHC: Immunohistochemistry
IP-WB: Immunoprecipitation and Western blot
KIRC: Kidney clear cell carcinoma
LIHC: Liver hepatocellular carcinoma
LUAD: Lung adenocarcinoma dataset
LUSC: Lung squamous cell carcinoma dataset
MBD: methyl-CpG binding domain proteins
MS-HRM: Methylation-sensitive high resolution melting analysis
NHBEC: Normal human bronchial epithelial cell
NL: Corresponding non-malignant lung tissue samples
NSCLC: Non-small cell lung cancer
NSG mice: NSG JAX (NOD.Cg-*Prkdc* ^*scid*^ *Il2rg* ^*tm1Wjl*^ */SzJ*) mice
OS: Overall survival
PRAD: Prostate adenocarcinoma
RNA-seq: IlluminaHiSeq RNA-sequencing
ROC: Receiver operating characteristic
RT-PCR: Real-time reverse transcription PCR
SNV: Single nucleotide variants
TCGA: “The Cancer Genome Atlas”
TSA: Trichostatin A
TSG: Tumor suppressor gene
TU: Primary tumor samples
