# Supplementary material for: SPAG6 and L1TD1 are transcriptionally regulated by DNA methylation in non-small cell lung cancers
Source: Mol Cancer. 2017 Jan 5;16:1. doi: 10.1186/s12943-016-0568-5 (PMC5240214; doi:10.1186/s12943-016-0568-5)
Supplement: Additional file 1: Table S1. — Description of NSCLC cell lines used in this study. Information about histology, origin and disease stage of donors was obtained from ATCC catalogue (https://www.lgcstandards-atcc.org). EGFR, KRAS and TP53 mutational status and MET amplification according to supplementary references (1–3). *activating EGFR mutation in exon 19 (E746-E749 del), **activating EGFR mutation in exon 21 (L858R). N/A, not available; wt, wildtype; mut, mutated. Table S2. Clinico-pathological characteristics of 983 NSCLC patients. Overview of gender, histology, stage of disease and ethnicity of NSCLC patients obtained from TCGA database and used for mutation and copy number changes analyses of SPAG6 and L1TD1 is shown. ADC, adenocarcinoma; SCC, squamous cell carcinoma. Clinical data based on Caleydo software version 16/04/14. Table S3. Primer sequences. Summary of oligonucleotide sequences used for mRNA expression, MS-HRM, BGS analyses and construction of pCMV6-GFP expression vector. Y, random integration of C or T in fwd primer; R, random integration of G or A in rev primer. Table S4. Methylation of SPAG6 and L1TD1 in tumor cells of other tumor types. *Morphology, histology and origin of cell lines according to ATCC catalogue (https://www.lgcstandards-atcc.org). Percentage of methylation was calculated as described previously (4). (DOCX 33 kb) [file 12943_2016_568_MOESM1_ESM.docx]

**Table S1. Description of NSCLC cell lines used in this study.**

| **Cell line** | **Histology** | **Origin** | **Disease stage** | ***EGFR*** | ***KRAS*** | ***TP53*** | ***MET* amplification** |
| --- | --- | --- | --- | --- | --- | --- | --- |
| A549 | Carcinoma | Primary, lung epithelial | N/A | wt | mut | wt | No |
| NCI-H1993 | Adenocarcinoma | Metastatic, lymph node | IIIA | wt | wt | mut | Yes |
| NCI-H1650 | Adenocarcinoma, bronchoalveolar carcinoma | Metastatic, pleural effusion | IIIB | mut* | wt | mut | No |
| NCI-H1975 | Adenocarcinoma | Primary, lung epithelial | N/A | mut** | wt | mut | No |
| HCC827 | Adenocarcinoma | Primary, lung epithelial | N/A | mut* | wt | wt | No |

Information about histology, origin and disease stage of donors was obtained from ATCC catalogue (https://www.lgcstandards-atcc.org). *EGFR*, *KRAS* and *TP53* mutational status and *MET* amplification according to supplementary references (1-3).*activating *EGFR* mutation in exon 19 (E746-E749 del), **activating *EGFR* mutation in exon 21 (L858R). N/A, not available; wt, wildtype; mut, mutated.

**Table S2. Clinico-pathological characteristics of 983 NSCLC patients.**

|  | **LUAD** | **LUSC** |
| --- | --- | --- |
| **Total** | **493** | **490** |
| **Gender** |  |  |
| Female | 262 | 122 |
| Male | 229 | 359 |
| NA | 2 | 9 |
| **Stage of disease** |  |  |
| I | 5 | 3 |
| IA | 124 | 80 |
| IB | 137 | 150 |
| IIA | 47 | 64 |
| IIB | 71 | 89 |
| IIIA | 70 | 64 |
| IIIB | 11 | 21 |
| IV | 25 | 7 |
| NA | 3 | 12 |
| **Ethnicity** |  |  |
| Caucasian | 388 | 294 |
| Asian | 7 | 9 |
| Afro-American | 29 | 17 |
| NA | 69 | 170 |

Overview of gender, histology, stage of disease and ethnicity of NSCLC patients obtained from TCGA database and used for mutation and copy number changes analyses of *SPAG6* and *L1TD1* is shown. ADC, adenocarcinoma; SCC, squamous cell carcinoma. Clinical data based on Caleydo software version 16/04/14.

**Table S3. Primer sequences.**

| **Gene** | **Primer sequence (5´ to 3´)** |
| --- | --- |
| **SYBR Green RT-PCR assay** |  |
| *L1TD1* fwd | CCAGCCAAAAATCTTTGTGAAAG |
| *L1TD1* rev | TCTCCAGCTCTATGCTTTGAGTC |
| *GAPDH* fwd | AGAAGGCTGGGGCTCATTTG |
| *GAPDH* rev | AGGGGCCATCCACAGTCTTC |
| **MS-HRM assay** |  |
| *SPAG6* fwd | GGGTTGTAATTTATTYGTTTGG |
| *SPAG6* rev | TATCCCTACACTACCCRAAAAT |
| *L1TD1* fwd | GGGGTAGTAATGGAAGTTAGTAAT |
| *L1TD1* rev | CTACTCCCCTCCTAAATATCC |
| **BGS analyses** |  |
| *SPAG6* fwd | AAGTTGTATTGATATTTATGTGTTGA |
| *SPAG6* rev | AAACCRCTTCTCTATATACCC |
| *L1TD1* fwd | GAGGTGATTTTGGGGTTTAG |
| *L1TD1* rev | CTAAACCAACCTTACCCAAAAC |
| **Vector construction** |  |
| *GFP* fwd | GAGGCGATCGCCATGGTGAGCAAGGGCG |
| *GFP* rev | GCGACGCGTCTTGTACAGCTCGTCCATG |

Summary of oligonucleotide sequences used for mRNA expression, MS-HRM, BGS analyses and construction of pCMV6-GFP expression vector. Y, random integration of C or T in fwd primer; R, random integration of G or A in rev primer.

**Table S4. Methylation of *SPAG6* and *L1TD1* in tumor cells of other tumor types.**

| **Cell line** | **Morphology, Histology*** | **Origin** | ***SPAG6 (%)*** | ***L1TD1 (%)*** |
| --- | --- | --- | --- | --- |
| MCF-7 | Epithelial, adenocarcinoma | Breast; derived from metastatic site: pleural effusion | 94 | 101 |
| MDA-MB453 | Epithelial, metastatic carcinoma | Breast; derived from metastatic site: pericardial effusion | 94 | 94 |
| MDA-MB468 | Epithelial, adenocarcinoma | Breast; derived from metastatic site: pleural effusion | 88 | 100 |
| MDA-MB231 | Epithelial, adenocarcinoma | Breast; derived from metastatic site: pleural effusion | 96 | 96 |
| BT20 | Epithelial, carcinoma | Breast; primary | 97 | 103 |
| HCT15 | Epithelial, adenocarcinoma | Colon, Dukes` type C; primary | 92 | 96 |
| HT29 | Epithelial, adenocarcinoma | Colon; primary | 87 | 95 |
| SK-OV3 | Epithelial, adenocarcinoma | Ovary, ascites; primary | 71 | 96 |
| A2780 | Epithelial, adenocarcinoma | Ovary; primary | 80 | 95 |
| AsPc1 | Adenocarcinoma | Pancreas; derived from metastatic site: ascites | 91 | 107 |
| BxPC3 | Epithelial, Adenocarcinoma | Pancreas; primary | 88 | 88 |
| CAL27 | Epithelial squamous cell carcinoma | Tongue; primary | 95 | 101 |
| FaDu | Epithelial, squamous cell carcinoma | Pharynx; primary | 99 | 113 |

*Morphology, histology and origin of cell lines according to ATCC catalogue (https://www.lgcstandards-atcc.org). Percentage of methylation was calculated as described previously (4).

**Supplementary references**

1. Li W, Mukherjee A, Wu J, Zhang L, Teves ME, Li H, et al. Sperm Associated Antigen 6 (SPAG6) Regulates Fibroblast Cell Growth, Morphology, Migration and Ciliogenesis. Sci Rep. 2015;5:16506.

2. Gong HC, Wang S, Mayer G, Chen G, Leesman G, Singh S, et al. Signatures of drug sensitivity in nonsmall cell lung cancer. Int J Proteomics. 2011;2011:215496.

3. Mahoney CL, Choudhury B, Davies H, Edkins S, Greenman C, Haaften G, et al. LKB1/KRAS mutant lung cancers constitute a genetic subset of NSCLC with increased sensitivity to MAPK and mTOR signalling inhibition. Br J Cancer. 2009;100:370-5.

4. Heller G, Babinsky VN, Ziegler B, Weinzierl M, Noll C, Altenberger C, et al. Genome-wide CpG island methylation analyses in non-small cell lung cancer patients. Carcinogenesis. 2013;34:513-21.
