# Supplementary figures and images for: SPAG6 and L1TD1 are transcriptionally regulated by DNA methylation in non-small cell lung cancers
Source: Mol Cancer. 2017 Jan 5;16:1. doi: 10.1186/s12943-016-0568-5 (PMC5240214; doi:10.1186/s12943-016-0568-5)

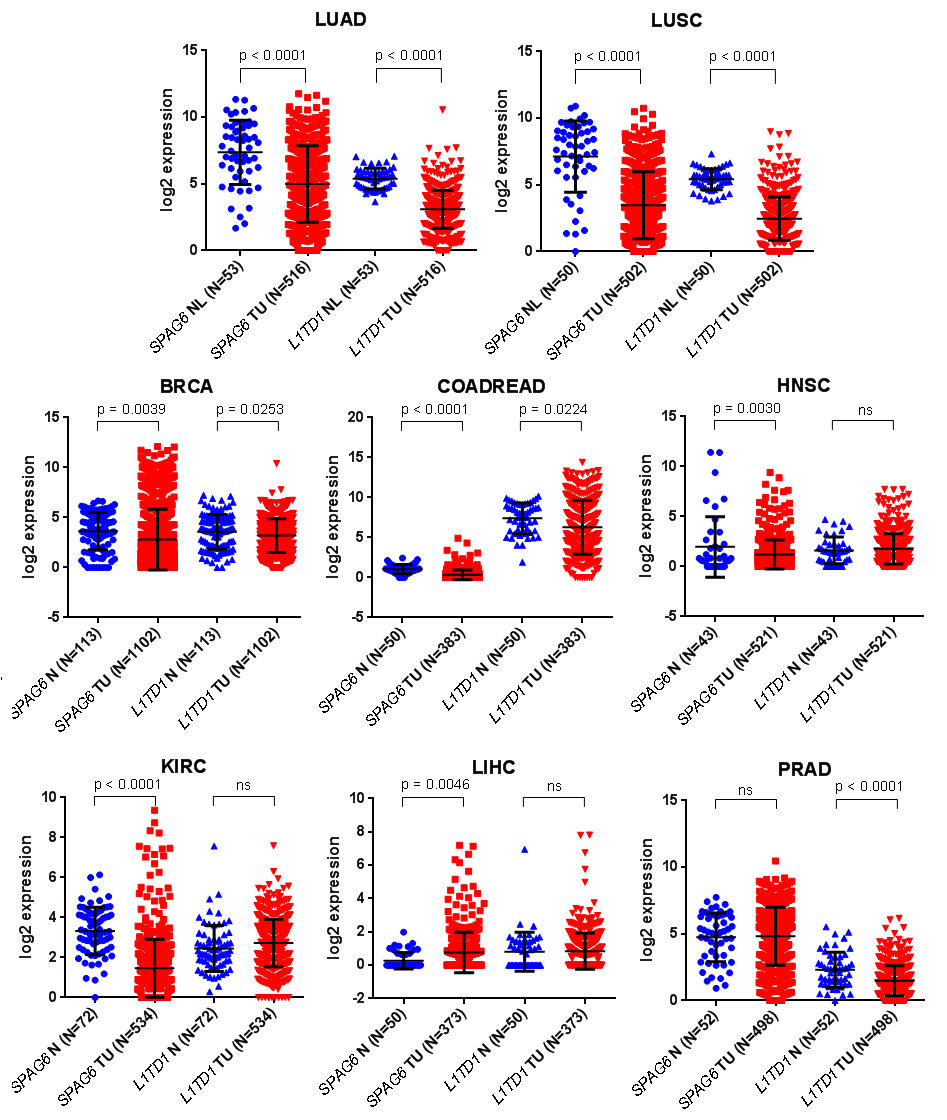

Supplement: Additional file 2: Figure S1. — SPAG6 and L1TD1 mRNA expression in different datasets of TCGA database. SPAG6 and L1TD1 mRNA expression was analysed using IlluminaHiSeq RNAseq data from TCGA database. Datasets LUAD and LUSC (lung), BRCA (breast), COADREAD (colorectal), HNSC (head and neck), KIRC (kidney), LIHC (liver) and PRAD (prostate) were analysed. Normalized log2 mRNA expression values are shown. Each dot represents a single sample. (TIF 176 kb) [file 12943_2016_568_MOESM2_ESM.tif]

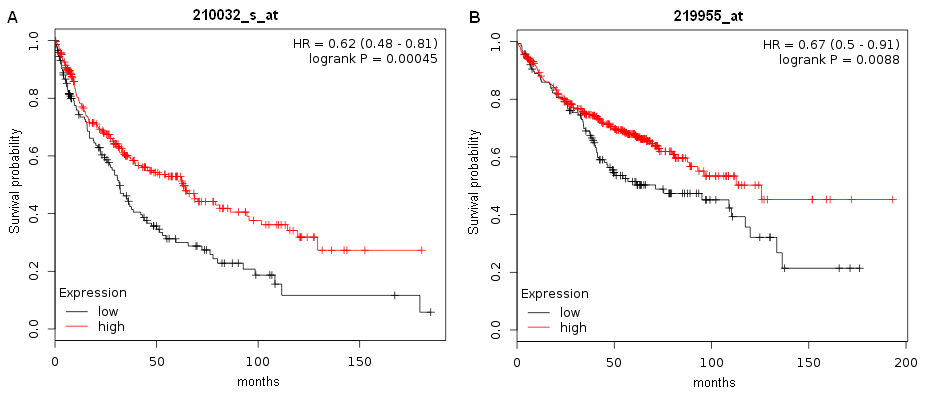

Supplement: Additional file 3: Figure S2. — Impact of SPAG6 and L1TD1 mRNA expression on OS of NSCLC patients. (A) A shorter OS of squamous cell carcinoma patients with low SPAG6 mRNA expression (N = 155) compared to high SPAG6 mRNA expression (N = 267) was observed. (B) Adenocarcinoma patients with low L1TD1 mRNA expression (N = 138) showed a shorter OS compared to adenocarcinoma patients with high L1TD1 mRNA expression (N = 350). Gene expression microarray datasets (Affymetrix IDs 210032_s_at and 219955_at) were analysed and Kaplan-Meier plots were generated using all datasets and default settings of KM plotter. The cut-off values for “low” and “high” SPAG6 and L1TD1 mRNA expression were automatically defined by KM plotter software (Version 2013). (TIF 50 kb) [file 12943_2016_568_MOESM3_ESM.tif]

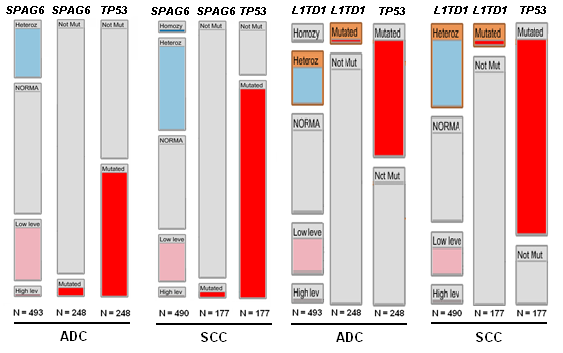

Supplement: Additional file 4: Figure S3. — SPAG6 and L1TD1 SNVs and deletions in NSCLC patients. TCGA LUAD and LUSC datasets were analysed with Caleydo software (version April 2014). Mutation of TP53 was used to demonstrate reliability of TCGA data analysis. ADC, adenocarcinoma patients; SCC, squamous carcinoma patients. (TIF 90 kb) [file 12943_2016_568_MOESM4_ESM.tif]

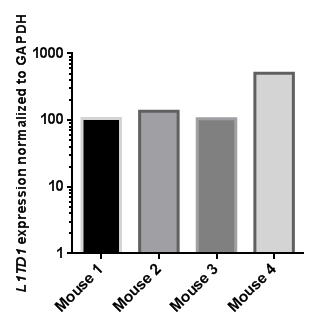

Supplement: Additional file 5: Figure S4. — L1TD1 mRNA expression in xenograft tumors. Expression of L1TD1 in 4 xenografts derived from pCMV6-L1TD1 transfected NCI-H1975 cells was confirmed by RT-PCR. GAPDH was used as housekeeping gene to normalize mRNA expression of L1TD1. (TIF 17 kb) [file 12943_2016_568_MOESM5_ESM.tif]
